# Relative Weight of Organic Waste Origin on Compost and Digestate 16S rRNA Gene Bacterial Profilings and Related Functional Inferences

**DOI:** 10.3389/fmicb.2021.667043

**Published:** 2021-05-14

**Authors:** Axel Aigle, Emilie Bourgeois, Laurence Marjolet, Sabine Houot, Dominique Patureau, Emmanuel Doelsch, Benoit Cournoyer, Wessam Galia

**Affiliations:** ^1^Univ Lyon, UMR Ecologie Microbienne (LEM), Université Claude Bernard Lyon 1, CNRS 5557, INRAE 1418, VetAgro Sup, Marcy L’Etoile, France; ^2^UMR ECOSYS, INRAE, AgroParisTech, Thiverval-Grignon, France; ^3^INRAE, Univ Montpellier, LBE, Narbonne, France; ^4^CIRAD, UPR Recyclage et risque, Montpellier, France; ^5^Recyclage et Risque, Univ Montpellier, CIRAD, Montpellier, France

**Keywords:** organic waste, anaerobic digestion, composting, 16S rRNA gene meta-barcoding, functional traits

## Abstract

Even though organic waste (OW) recycling via anaerobic digestion (AD) and composting are increasingly used, little is known about the impact of OW origin (fecal matters and food and vegetable wastes) on the end products’ bacterial contents. The hypothesis of a predictable bacterial community structure in the end products according to the OW origin was tested. Nine OW treatment plants were selected to assess the genetic structure of bacterial communities found in raw OW according to their content in agricultural and urban wastes and to estimate their modifications through AD and composting. Two main bacterial community structures among raw OWs were observed and matched a differentiation according to the occurrences of urban chemical pollutants. Composting led to similar 16S rRNA gene OTU profiles whatever the OW origin. With a significant shift of about 140 genera (representing 50% of the bacteria), composting was confirmed to largely shape bacterial communities toward similar structures. The enriched taxa were found to be involved in detoxification and bioremediation activities. This process was found to be highly selective and favorable for bacterial specialists. Digestates showed that OTU profiles differentiated into two groups according to their relative content in agricultural (manure) and urban wastes (mainly activated sludge). About one third of the bacterial taxa was significantly affected by AD. In digestates of urban OW, this sorting led to an enrichment of 32 out of the 50 impacted genera, while for those produced from agricultural or mixed urban/agricultural OW (called central OW), a decay of 54 genera over 60 was observed. Bacteria from activated sludge appeared more fit for AD than those of other origins. Functional inferences showed AD enriched genera from all origins to share similar functional traits, e.g., chemoheterotrophy and fermentation, while being often taxonomically distinct. The main functional traits among the dominant genera in activated sludge supported a role in AD. Raw OW content in activated sludge was found to be a critical factor for predicting digestate bacterial contents. Composting generated highly predictable and specialized community patterns whatever the OW origin. AD and composting bacterial changes were driven by functional traits selected by physicochemical factors such as temperature and chemical pollutants.

## Introduction

Organic wastes (OWs) have increased 10-fold since the last century and are likely to double by 2025 as a result of the growth of the world population (Hoornweg et al., 2013). There is thus a strong need for sustainable OW management. From a waste management standpoint, aerobic (composting) and anaerobic digestions (AD) are the most obvious operational processes prior to soil applications. These recycling treatments can also be combined by performing a composting of digestates. A major constraint in these OW recycling scenarios is the capacity to assess the related OW health hazards, which are, in fact, dependent on their origin and contents in terms of chemical and microbiological contaminants, and the efficacy of the treatments at reducing these hazards.

AD implies a breakdown of organic materials naturally found in raw OW into biogas (50–75% CH_4_, 25–50% CO_2_, and 1–2% H_2_S, H_2_, and NH_3_) and organic residues (called digestate) by a microbial consortium (Atelge et al., 2020). The success of this process relies on the synergies between microorganisms acting in a coordinated succession driven by the environmental changes occurring during the process (Rivière et al., 2009; Yu et al., 2010). To avoid deviation of microbial communities during the AD process, parameters such as volatile fatty acids (VFAs), ammonia and hydrogen sulfide concentrations, acetate:propionate ratios, and pH are regularly monitored (Kayhanian, 1994; Chen et al., 2008; González-Fernández and García-Encina, 2009; Wang et al., 2009; Franke-Whittle et al., 2014; Wagner et al., 2014). The microbial reactions of these processes can be performed by psychrophiles, mesophiles, and thermophiles (Moset et al., 2015; Saady and Massé, 2015; Hupfauf et al., 2018). Mesophilic digestion is considered the most stable AD process (Gou et al., 2014) because diversity of bacterial and archaeal assemblages are greater at mesophilic (35–37°C) temperatures (Levén et al., 2007; Pycke et al., 2011). The initial hydrolysis step of OW is performed by many facultative anaerobes or strictly anaerobic bacteria. This hydrolysis can degrade complex molecules into simple sugar monomers, amino acids, and alcohols. Fermentative bacteria will then convert these monomers into short-chain fatty acids, primary alcohols, as well as H_2_ and CO_2_. The production of acetate from the fatty acids will be performed by homoacetogenic bacteria like *Butyribacterium* and *Acetobacterium*, that of H_2_ will be performed by the acetogenic microbiota such as *Syntrophomonas*, and that of CO_2_ and H_2_S will be performed by sulfate-reducing bacteria such as *Desulfovibrio* and *Desulfobacter*. Some of these compounds will then be used by methanogens to generate CH_4_. Hydrogenotrophic methanogens such as *Methanobacterium* and *Methanogenum* sp. convert H_2_ and CO_2_ into CH_4_, and acetotrophic ones such as *Methanosarcina* will convert acetate into CH_4_ (Demirel and Scherer, 2008; Lykidis et al., 2011).

In the same way, composting is a natural process characterized by the conversion of OW into by-products that can be used as soil conditioners and/or organic fertilizers (Reinikainen and Herranen, 2001; Ahmad et al., 2007). This process is performed by an aerobic microbial consortium composed of *Proteobacteria*, *Actinobacteria*, and fungi. During the process of composting, microbial community successions are driven by environmental parameters such as moisture, temperature, and the aeration conditions (Blanc et al., 1999; Tian et al., 2013; Zhang et al., 2016). Composting in its initial phase can be carried out by several mesophiles including bacteria, which represent environmental health hazards such as *E. coli*, *Salmonella*, *Klebsiella*, and *Nocardia* (Insam and de Bertoldi, 2007). These organisms can break down soluble organic compounds by exothermic reactions. As the temperature rises above 40°C, these mesophiles will be outcompeted by more thermophilic bacteria such as *Bacillus stearothermophilus* and species from the *Deinococcus*/*Thermus* group (Insam and de Bertoldi, 2007). These will continue the breakdown processes and generate higher temperatures that will partly sanitize the end products by killing heat-sensitive hazardous microbial agents. A compost pile can reach 65–70°C. As the supply of high-energy compounds will get low, thermo-tolerant mesophiles will be favored and will perform the last transformation or “stabilization” reactions (Day and Shaw, 2001). The cool zones in the composting systems, particularly with those having no or little mixing, can favor a survival and (re)growth of some bacterial pathogens (Noble and Roberts, 2004).

Beyond the general microbial structures described previously for AD and composting, several questions remain, including how the high inputs of microorganisms and cells from the feeding substrates (OW) under conditions of continuous flow impact the microbial community structure in the end products. On one hand, some studies suggested that the microbial communities of digested OW are unique to a digester because of high differences in substrate and operating conditions (Werner et al., 2011; Sundberg et al., 2013; Zhang et al., 2014; De Vrieze et al., 2016). However, some of these analyses on full-scale AD plants fed with a mixture of substrates (De Vrieze et al., 2016) or with activated sludge (Wang et al., 2018) also indicated that the microbial communities could be stable over time. Nevertheless, in other studies, the microbial communities in AD were found variable over time (among a same digester), even when substrate and operational conditions remained constant (Supaphol et al., 2011; De Vrieze et al., 2013; Williams et al., 2013; Vanwonterghem et al., 2014). On the other hand, during composting, microbial communities of the raw OW were found to trigger a rise and decrease in temperature (Palaniveloo et al., 2020). These changes are highly selective but their incidence on microbial community patterns of mature compost remain to be evaluated according to the origin of OW. A knowledge gap thus remains about the relative contribution of the raw OW microbiome on the digestate and compost structural and functional microbial community patterns.

Here, we made the hypothesis that the microbiomes found among digesters and composts will be (i) significantly structured by the communities of the raw OW inputs, but that (ii) functional redundancy among these communities would generate distinct community profiles between treatment plants. Similar biodegradation activities between distinct treatment plants would thus be achieved by different microbial organizations because of this functional redundancy. To test these hypotheses, nine full-scale treatment plants located in various rural and urban backgrounds were investigated. These treatment plants were fed with agricultural or urban wastes or a mixture of both. Bacterial genetic structures were inferred from 16S rRNA gene meta-barcoding approaches, and patterns were compared between process types and according to the origin and nature of the raw OW. Functional traits were inferred from the 16S rRNA gene taxonomic allocations and databases recording the metabolic processes of well-defined bacterial taxa (FAPROTAX). The theoretical metabolic pathways of the genera impacted by treatment were further inferred from sequenced genomes using the MACADAM software (Le Boulch et al., 2019).

## Materials and Methods

### OW Recycling Sites and Samplings

Nine full-scale OW treatment plants (numbered as Urban-1-2-3, Agri-1-2-3 and Central-1-2-3) were considered in this study (Table 1). The sampling strategy implied the use of at least three independent treatment plants per sample categories. OWs were treated by AD (Urban-1-2, Agri-1-2, and Central-1-2-3) or composting (Urban-1-3 and Agri-1-3). Five of the AD plants applied a post-treatment to the digestate: solid/liquid separation (Central-1-2), solid/liquid separation followed by composting (Urban-1 and Agri-1), or solid/liquid separation followed by solid phase drying (Urban-2). Raw OWs were chosen to represent the most common origins of OW: urban [sewage sludge produced by wastewater treatment plants (WWTP): Urban-1-2-3], agricultural (exclusively or mainly composed of animal manure and/or slurry: Agri-1-2-3), and central (a mix of food industry waste, harvest residues, livestock effluents, and/or sewage sludge: Central-1-2-3) (see Table 1 for details of the OWs compositions). For all sites, samples were taken at each treatment step to capture treatment effects on the microbial community’s composition and abundance. Thus, a raw OW sample was taken from each site, resulting in nine samples, respectively, defined as urban (Urban-1-2-3-OW), agricultural (Agri-1-2-3-OW), and central (Central-1-2-3-OW) (Table 1). Digestates were available and sampled for six sites (Urban-1-D, Agri-1-2-D, and Central-1-2-3-D), as well as solid and dried digestates, respectively, from liquid/solid phase separation, and from solid phase drying (Urban-1-2-SD, Agri-1-SD, Central-1-2-SD, and Urban-2-DD) (Table 1). Liquid digestates were available for four sites (Urban-2-LD, Agri-1-LD, and Central-1-2-LD), and finally, compost was produced and sampled also at four sites (Urban-1-3-C and Agri-1-3-C) (Table 1). At least 1 kg of OW, composts, or digestates was collected per treatment. Samples were coded as indicated in Le Bars et al. (2018). For each sample, certain pollutants and physicochemical parameters were measured (Supplementary Tables 1, 2). These datasets have been presented in Le Bars et al. (2018) and Sertillanges et al. (2020) and further analyzed in order to verify the chemical differentiation of samples according to their origin (urban, agricultural, and central). Total nitrogen (ISO 13878) and total carbon (ISO 10694) were determined by dry combustion with an elemental NC 2100 Soil Analyzer (Thermo Electron Corp.). Inorganic carbon (ISO 10693) was determined with a Bernard Calcimeter by adding HCl and measuring released CO2. Organic carbon (C_*org*_) was deduced from the difference between total and inorganic carbon. Concentrations of trace elements (TE) were measured using an inductively coupled plasma mass spectrometer (ICP-MS iCAP Q Thermo Fisher Scientific). pH was directly measured on liquid samples, or according to the ISO 10390. More details for those procedures are described in Le Bars et al. (2018). The concentrations of all the elements are expressed on a dry matter (DM) basis. Nonylphenols (NPs) and polycyclic aromatic hydrocarbons (PAHs) were measured in 1 g subsample according to Trably et al. (2004) and Mailler et al. (2017). More details for these monitorings are given in Sertillanges et al. (2020).

**TABLE 1:** Organic waste treatment plants and sample features.

Sampling sites	Reactor size (m^3^)/temperature for AD	Samples	Duration^*a*^/treatment^*b*^	Sample code	Organic waste composition and co-substrate used for composting
Urban-1 French wastewater treatment plant and composting platform	6,200 32–42°C	Organic waste Digestate Solid digestate Compost^*c*^	38d/AD 4w/C	Urban-1-OW Urban-1-D Urban-1-SD Urban-1-C	Drained activated sludge Co-substrate: green waste
Urban-2 French wastewater treatment plant	6,000 32–42°C	Organic waste Liquid digestate Solid digestate Dried digestate	34d/AD	Urban-2-OW Urban-2-LD Urban-2-SD Urban-2-DD	Drained sludge
Urban-3 French wastewater treatment plant and composting platform		Organic waste Compost^*d*^	6w/C	Urban-3-OW Urban-3-C	Dehydrated activated sludge Co-substrate: green waste
Agri-1 Anaerobic digestion plant and composting platform (France)	950 32–42°C	Organic waste Digestate Liquid digestate Solid digestate Compost^*c*^	38d/AD 12w/C	Agri-1-OW Agri-1-D Agri-1-LD Agri-1-SD Agri-1-C	Porcine slurry (for 6–7 T/day) and grass clippings, food and vegetable wastes, corn silage and straws Co-substrate: none
Agri-2 Anaerobic digestion plant (France)	740 45°C	Organic waste Digestate	42d/AD	Agri-2-OW Agri-2-D	80% of bovine manure and 20% of stercoral material, poultry litter, corn cob, cooking oils, lawn mowing and bran
Agri-3 Composting platform (France)		Manure Compost^*d*^	12w/C	Agri-3-OW Agri-3-C	Raw cattle, sheep and equine manures Co-substrate: none
Central-1 Anaerobic digestion plant (France)	1,600 32–42°C	Organic waste Digestate Liquid digestate Solid digestate	70d/AD	Central-1-OW Central-1-D Central-1-LD Central-1-SD	50% livestock manures (N_manure + animal slurry), 28% wastewater treatment plant sludge, 22% food industry by-products
Central-2 Anaerobic digestion plant (France)	6000 32–42°C	Organic waste Digestate Liquid digestate Solid digestate	30d/AD	Central-2-OW Central-2-D Central-2-LD Central-2-SD	45% pig slurry, 45% wastes from food industries, 10% cereal mixture
Central-3 Anaerobic digestion plant (France)	8,000 32–42°C	Organic waste Digestate	90d/AD	Central-3-OW Central-3-D	50% biowastes (community, catering, mass distribution), 20% dairy cow slurry, 30% wastes from food industries and agricultural by-products

*^*a*^Process duration (d = days/w = weeks). ^*b*^Treatments (AD = anaerobic digestion/C = composting). ^*c*^The substrates used for composting were solid digestates from the matching site (Urban-1 or Agri-1). ^*d*^Raw organic wastes were used for the composting at the Urban-3 and Agri-3 sites.*

### DNA Extractions and MST

DNA was extracted from 0.5 g of solid samples or 500 μl of liquid samples using the FastDNA Spin kit for soil and the FastPrep-24 (MP Biomedicals) following the manufacturer’s instructions. All the DNA extractions were triplicated for each sample. DNA was eluted in 50 μl of ultrapure water, and quantity and quality were measured using a nanodrop-One UV-Vis Spectrophotometer (Thermo Fisher Scientific). DNA extracts were visualized after electrophoresis at 6 V/cm using a TBE buffer [89 mM Tris-borate, 89 mM boric acid, and 2 mM EDTA (pH 8.0)] through a 0.8% (w/v) agarose gel, and DNA staining with 0.4 mg ml^–1^ ethidium bromide. A Gel Doc XR+ System (Bio-Rad, France) was used to observe the stained DNA and confirm their relative quantities and qualities. DNA was kept at −80°C and shipped on ice within 24 h to the DNA sequencing services when appropriate.

MST methods were used to monitor host fecal contaminations by human, bovine, and pig (Supplementary Table 3). These tests were performed on all DNA extracts including all replicates from the same sample. MST qPCR amplifications of general and host-specific *Bacteroides* markers, as well as general 16S rRNA gene qPCR assays, were performed as described in Marti et al. (2017) and Voisin et al. (2020). The human-specific marker (HF183) and the 16S rRNA genes were quantified by the SYBR Green chemistry while the bovine-specific, pig-specific, and the total *Bacteroidetes* markers were quantified using the Taqman chemistry. Six qPCR assays were performed per sample using DNA obtained from three independent extractions (Supplementary Table 4). Standard curves were generated using 10-fold serial dilutions (ranging from 10^5^ to 10^0^ gene copies) of cloned targeted sequences (Marti et al., 2017). MST qPCR datasets were expressed per copy number of DNA targets per gram of dry sample and normalized by dividing their numbers by the 16S rRNA gene copy numbers per gram of dry sample.

### V5–V6 16S rRNA Gene Sequencing and Reads Processing

The V5–V6 region of the 16S rRNA genes were amplified using the 799F forward (barcode + ACCMGGATTAGATACCCKG) and 1193R reverse (CRTCCMCACCTTCCTC) primers (Chelius and Triplett, 2001; Bodenhausen et al., 2013; Supplementary Table 3) by the Molecular Research DNA lab (MrDNA, Shallowater, United States) and were sequenced by the Illumina MiSeq V3 technology. PCR amplifications were performed on pooled DNA extracts obtained per sample (equimolar mixes of the triplicates) in order to handle variability between DNA extracts. The HotStarTaq Plus Master Mix Kit (Qiagen, United States) using the following temperature cycles was used to generate the PCR products: 94°C for 3 min, followed by 28 cycles of 94°C for 30 s, 53°C for 40 s, and 72°C for 1 min, with a final elongation step at 72°C for 5 min. PCR products and blank control samples were verified using a 2% agarose gel and following the electrophoretic procedure described above. PCR products obtained from field samples showed sizes around 430 bp but blanks did not show detectable and quantifiable PCR products. Dual-index adapters were ligated to the PCR fragments using the TruSeq^®^ DNA Library Prep Kit, which also involved quality controls of the ligation step (Illumina, Paris, France). Illumina Miseq DNA sequencings of the PCR products were paired-end and set up to obtain around 40K reads per sample.

The generated sequences were processed with the Mothur package (Schloss et al., 2009) according to a standard operation protocol (SOP) for MiSeq-based microbial community analysis (Schloss et al., 2009; Kozich et al., 2013), so-called MiSeq SOP, and available at http://www.mothur.org/wiki/MiSeq_SOP. Briefly, primers and barcodes were trimmed according to quality and size filtration, followed by sequence alignment. Size filtration was refined, and unique sequences were extracted. The pre.cluster command was applied in order to mask the remaining sequencing errors as well as the split.abund command where the cutoff was set to 1. Chimera removal was performed using the chimera.uchime command and chimeric sequences were removed. At this stage, sequences were processed following two normalization strategies: (i) subsampling according to the sample containing the lowest number of sequences, and (ii) no subsampling (see statistical analysis for normalization strategy). Finally, for both datasets, the SILVA 16S rRNA Bacterial reference library was used for taxonomic allocation of the OTUs (cutoff 80)^1^ and OTU clustering was performed with a similarity threshold of 97% for subsampled reads (Supplementary Table 5) or using the phylotype command at the genus level, for non-subsampled reads (Supplementary Table 6). The Mothur package was also used to calculate diversity indices (Shannon, Simpson, evenness, and richness) on the 16S rRNA subsampled OTU table (Supplementary Table 7).

### Statistical Analyses

Relations between sample groupings and chemical datasets were tested by principal component analysis using the rda function and plotted using the biplot function; confidence ellipses were drawn to show the variance observed per group. Kruskal–Wallis tests were performed using the kruskal.test function on 16S rRNA diversity indices, MST markers ratio, and physical–chemical data. When significant differences were found (*p* < 0.05), a Dunn test was applied using the kwAllPairsDunnTest function from the PMCMRplus package (Pohlert, 2020) for *post hoc* analysis.

Hellinger transformation was applied on the 16S rRNA subsampled OTU table using the decostand function (method = “total”), and Bray–Curtis dissimilarities were computed between pairs of samples using the vegdist function (“bray”). NMDS was generated using the metaNMDS function. Ordiellipse option was used to display the variance observed per group, and PERMANOVA tests were performed using the pairwise.adonis and vegdist functions (Bray–Curtis dissimilarities) to confirm the significance of differences between groups.

The non-subsampled 16S rRNA gene reads genus table (Supplementary Table 6) was processed with the DESEQ2 package (Love et al., 2014) in order to reveal genera with significant change in abundance before and after treatment. Counts normalized by the median of ratios method (Anders and Huber, 2010) were recovered and relative abundances were calculated in order to present the percentage of significant differences in abundances at the genus level before and after treatment. Change in abundance has been considered significant if a genus shows a log2 fold change ≤ -2 or ≥ +2 with a minimum Base Mean value of 10 and adjusted *p* value of < 0.05. To facilitate global comparison with other studies, computations were also performed at the phylum level. All the functions used without package indication were from the vegan package (Oksanen et al., 2019) and all analyses were performed on RStudio (R v3.3.3). The statistical experimental design (Kruskal–Wallis tests on 16S rRNA diversity indices and MST markers, as well as NMDS and DESEQ2 analyses) implied first testing relations according to treatment (AD against composting), regardless of the OW origin and regardless of the post-treatments. Thus, all OW samples were compared to composted samples and digestates. Second, the OW origins were considered in the comparisons to infer their impact on the V5–V6 16S rRNA gene datasets.

The 16S rRNA gene sequences produced in this study are available from webin under the accession number PRJEB40193.

## Results

### General Chemical and Microbiological Features of the Raw OWs, Digestates, and Composts

**FIGURE 1:**
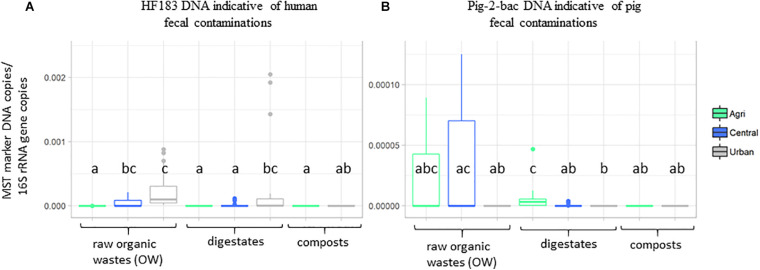
Boxplots showing the relative occurrences of two DNA markers indicative of human and pig fecal contaminations. Datasets are from Supplementary Table 4. Numbers of **(A)** HF183 and **(B)** pig-2-bac DNA copies per gram dry weight organic wastes, composts, or digestates normalized to 16S rRNA gene copy numbers were presented according to OW origins (agricultural, urban, and central). Boxplots show the minimum, first quartile (Q1), median, third quartile (Q3), and maximum relative abundances observed among the datasets. Outliers or unexpected values are indicated by dots. Samples used in these analyses are those of Table 1.

Principal component analyses were performed on the physicochemical datasets (organic carbon, total nitrogen, C:N ratio, pH, and phosphorus) and pollutants (iron and zinc concentrations, PAHs, NPs, and TE) measured on all samples (Supplementary Tables 1, 2 and Supplementary Figure 1). The first two axes of these PCAs explained a significant part of the variability observed among these datasets (>60%) (Supplementary Figure 1). The origin of the sample was found to explain the chemical dataset (Supplementary Figure 1). The urban samples (treated or not) were significantly differentiated from the agricultural and central (mixed) samples by a set of pollutants including several TE (Ag, Al, As, Cd, Ce, Co, Cr, Cu, Ga, K, Mo, Ni, P, Pb, and Ti), 4-nonylphenol (A2), phenanthrene (H5), anthracene (H6), and total nitrogen concentrations (KW–Dunn’s *post hoc* tests; *p* < 0.05) (Supplementary Figure 2). Similarly, global indicators of the microbiological content of the samples were analyzed by qPCR using microbial source tracking DNA targets (Supplementary Table 4 and Figure 1). These qPCR analyses showed that 16S rRNA gene numbers varied from 4.26 × 10^5^ copies/g sample up to 6.41 × 10^11^ copies/g (Supplementary Table 4). Lowest 16S rRNA gene copy numbers/g sample were obtained for digestates. Monitorings of total *Bacteroides* cell numbers were correlated with the 16S rRNA gene dataset (Kendall’s rank correlation tau; *p* < 0.01) but the range of values was lower (2.44 × 10^2^ copies/g sample up to 1.12 × 10^9^ copies/g). The DNA indicators for pig and bovine fecal contaminations were most significant among central and agricultural raw OW (Figure 1 and Supplementary Table 4). These distributions were in agreement with the information given by the operators of the OW treatment sites. The human DNA fecal marker was most often detected among the raw OW but in significantly higher numbers (KW and Dunn’s *post hoc*; *p* < 0.05) among the urban and central groups of samples (Supplementary Table 4 and Figure 1). Digestates and composts showed lower occurrences of these markers but a significant number of HF183 DNA was detected among the urban digestates (Figure 1).

### Bacterial Diversity of Raw OWs, Digestates, and Composts Inferred From V5 to V6 16S rRNA Gene Sequences

About 5 million DNA sequences were obtained for the samples investigated in this work, and 45% were validated as high-quality 16S rRNA gene sequences. The number of high-quality V5–V6 16S rRNA gene sequences per sample ranged from 26,465 to 119,793 (Supplementary Table 7). The number of reads per sample were negatively correlated to the 16S rRNA gene qPCR dataset (Spearman Rank Correlation; rho = −0.45, *p* < 0.05). This highlighted a variability in the dataset that required a normalization of the 16S rRNA gene dataset prior to performing statistical analyses. The global representation of the dataset and the computing of diversity indices were thus performed after a subsampling of the gene reads at the lowest value for the number of reads on a sample, i.e., 26,465 reads. This subsampled dataset led to a grouping of reads into 7755 OTUs, which varied in their distribution from 318 to 1404 per sample (Supplementary Table 5). Accordingly, Shannon diversity, Shannon-evenness, and the Simpson diversity indices were computed (Supplementary Table 7 and Supplementary Figure 3). Significant differences in these indices were observed between those computed for the urban digestates and those of the agricultural or central samples (KW test; *p* < 0.05). More diverse OTU contents and even distributions of the reads between OTUs were observed among the urban digestates than the other ones. However, the initial OTU contents of raw OW were found similar between sites, and diversity indices remained similar between raw OW and digestates of a same site (Supplementary Figure 3).

**FIGURE 2:**
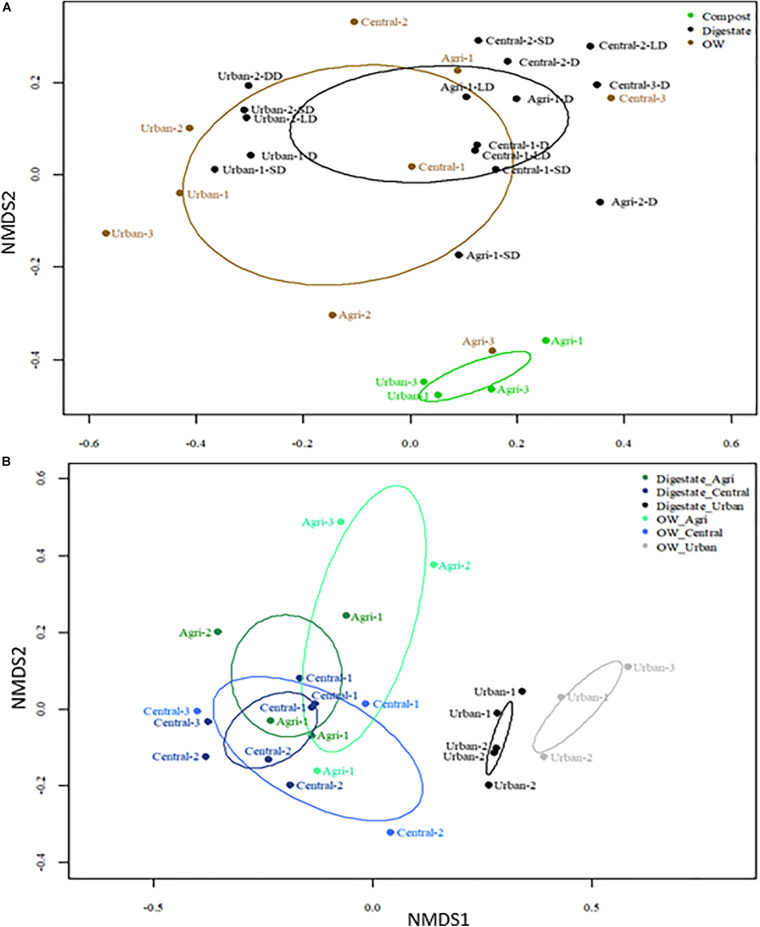
Non-metric multidimensional scaling (NMDS) plot of Bray–Curtis dissimilarities computed from the 16S rRNA V5–V6 OTU profiles (subsampled, 97% identity) of the organic samples before (raw OW) and after treatment by anaerobic digestion or composting. **(A)** NMDS ordinations were built from the full dataset shown in Table 1; stress value was 0.1227. **(B)** NMDS ordinations were built from a dataset limited to raw OW and digestates. The ellipses indicate the variance observed within each group of samples. PERMAMOVA tests are indicated in the text and confirmed the significance (*p* < 0.05) of several groupings.

To better resolve differences in the genetic diversity among samples, Bray–Curtis dissimilarities between OTU profiles of each pair of samples were computed. NMDS plots were then performed to visualize the differences (Figure 2A). All OTU profiles could be differentiated according to their site category (urban, central, or agricultural) (PERMANOVA *p* < 0.05). A significant effect on the profiles of the OW treatment by composting or AD could also be resolved (PERMANOVA *p* < 0.05). Digestates’ OTU profiles could not be differentiated into three categories (PERMANOVA *p* > 0.05) but into two entities with (i) grouping the agricultural/mixed central samples and (ii) the urban ones (PERMANOVA *p* < 0.01) (Figure 2B). A further differentiation of OTU profiles according to post-treatments of the digestates was tested. The OTU patterns of liquid and solid digestates could not be differentiated (Supplementary Figure 4). Composting was found to drive bacterial community changes toward similar structures even though the nature of the OWs was variable (WWTP activated sludge, manure, green wastes, etc.) (PERMANOVA *p* < 0.05) (Figure 2A).

### Incidence of AD or Composting on Bacterial Taxa as Inferred From 16S rRNA Gene Taxonomic Allocations

The chemical and V5–V6 16S rRNA gene OTU pattern analyses indicated in the previous sections showed (i) a significant proximity between the agricultural and central samples (raw OW and digestates), (ii) a significant differentiation of the urban samples (raw OW and digestates) from the agricultural/central samples, and (iii) a significant differentiation of the compost samples from all the other samples. Accordingly, in the following analyses, the datasets were grouped into these latter three entities. Informative groupings of V5–V6 DNA reads matching significant changes (higher or lower) in relative abundances between these entities were analyzed.

#### Variations at the Scale of Phyla

**FIGURE 3:**
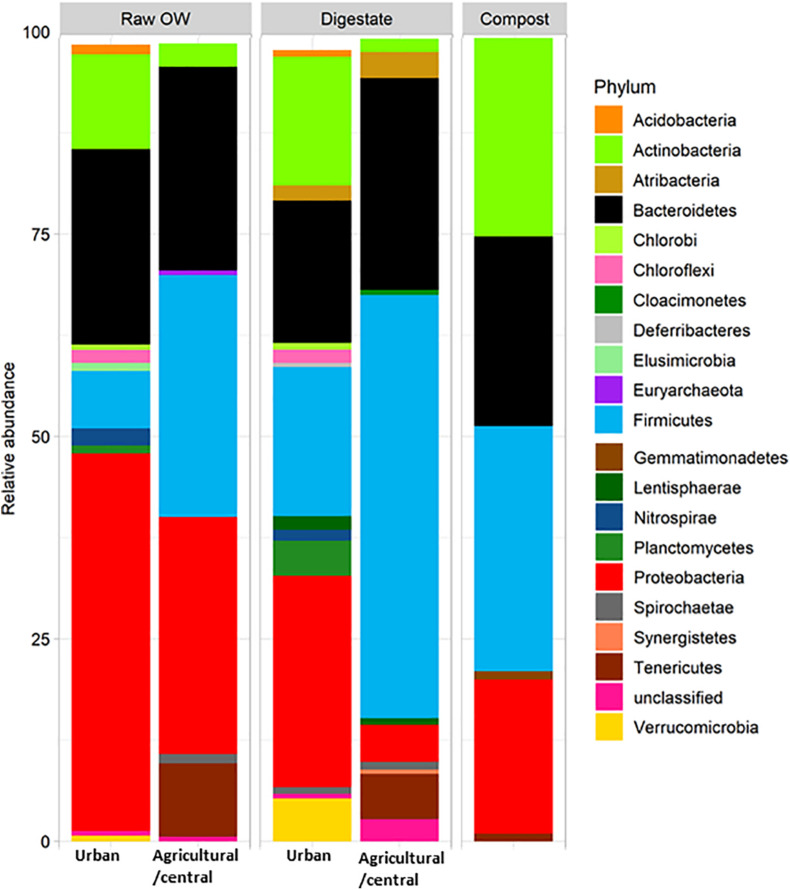
Relative averaged abundances of V5–V6 16S rRNA gene reads allocated to phyla before and after treatment of raw organic wastes (OWs) by anaerobic digestion or composting performed by treatment plants located in rural areas (agricultural/central sites) or urban ones. Only phyla with a relative abundance above 0.5% are displayed (see Supplementary Table 9 for the complete dataset).

Raw OWs showed a high variability in their OTU contents (Figure 2A). However, these OTUs can be grouped into larger taxonomic entities, which can be conserved between raw OWs, and contribute reproducibly to the buildup of the microbial communities among composts or digestates. These core taxa were found to be distributed among 6 major phyla: *Firmicutes* (31.2%), *Proteobacteria* (26.4%), *Bacteroidetes* (26.2%), *Actinobacteria* (6.1%), *Tenericutes* (4.4%), and *Spirochaetae* (0.7%) but discrepancies were observed in terms of proportion of reads per phylum according to the site category (urban, central, and agricultural) (Supplementary Table 8). Raw urban OW showed lower relative numbers (7.1%) of *Firmicutes* [converted into 2.05 × 10^9^ equivalent 16S rRNA gene copy numbers (16S) per gram of OW] than the raw OWs from the central and agricultural sites (29.9%; equivalent to 1.01 × 10^10^ 16S per gram) (Supplementary Table 9 and Figure 3). These urban OWs were found to have higher relative counts of *Proteobacteria* (46.6%; equivalent to 3.97 × 10^10^ 16S per gram) and *Actinobacteria* (11.7%; equivalent to 6.51 × 10^9^ 16S per gram) than the central and agricultural samples (respectively, 29.3%; equivalent to 8.74 × 10^9^ 16S per gram and 2.9%; equivalent to 1.2 × 10^9^ 16S per gram) (Supplementary Table 9 and Figure 3). It is to be noted that 16S rRNA gene reads allocated to the *Nitrospirae*, *Chloroflexi*, and *Acidobacteria* represented more than 1% (equivalent to 7.41 × 10^9^ 16S rRNA gene copies per gram) of the urban OW dataset, but these phyla occurred at a much lower score (equivalent to 4.32 × 10^6^ 16S rRNA gene copies per gram) among the OW of the other sites (Supplementary Table 9 and Figure 3).

As observed among raw OW, *Firmicutes* and *Proteobacteria* were among the most abundant phyla in digestates and composts (Supplementary Table 9 and Figure 3). However, a decrease in the relative abundance of the *Proteobacteria* was observed in all treated samples (Supplementary Table 9 and Figure 3) and was supported by a significant statistical test for the urban samples (KW *p* ≤ 0.05). These changes in proportion were not correlated to a significant reduction in bacterial cell numbers (between 1.77 × 10^9^ and 3.19 × 10^10^ 16S rRNA gene copies per gram of digestates or composts). The observed change in relative abundance thus implies a significant re-shuffling of bacterial phyla with some of these taking advantage of the treatment conditions or becoming negligible. For example, increases in the relative proportion of *Firmicutes* from the raw OW to the digestates were observed among both categories of samples (agricultural/central, and urban). *Firmicutes* showed the highest occurrence among the central/agricultural digestates (52.2% of the bacterial taxa). These relative changes matched an averaged one log increase in *Firmicutes* 16S rRNA gene copies between the raw OW and the digestates of the urban sites or composts (Supplementary Table 9 and Figure 3). Similarly, relative increases in *Actinobacteria*, *Deferribacteres*, and *Thermotogae* among composts matched significant increases in 16S rRNA gene copies estimated from the qPCR datasets (Supplementary Table 9 and Figure 3; KW *p* ≤ 0.05). Similar trends were also observed between raw OW and urban digestates for the *Cloacimonetes*, which showed up to a five-log increase in 16S rRNA gene copies per gram. Significant decreases in the relative abundance of some phyla were also recorded such as the raw OW *Fusobacteria*, which became negligible among the digestates and composts (Supplementary Table 9 and Figure 3; KW *p* ≤ 0.05).

#### Variations at the Scale of Genera Among Digestates

**FIGURE 4:**
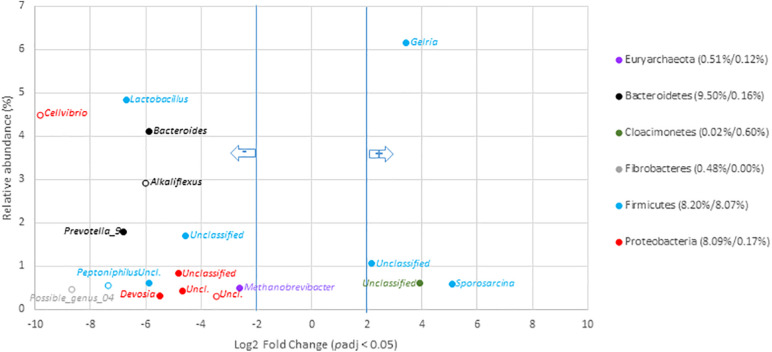
Genera with significant changes in relative abundance after anaerobic digestion of raw organic waste samples from agricultural and central mixed origin. Genera with significant (*p*_*adj*_ < 0.05) decrease in digestates compared to raw OW samples (left part of the plot) are presented according to their initial relative abundance in raw OW. Genera with significant increase in digestates compared to raw OW samples (right part of the plot) are presented according to their relative abundance in digestate. Genera are colored according to the phylum to which they belong. Full mark represents genus present in the core microbiome; empty mark represents genus not present in the core microbiome (raw OW and digestate core microbiomes were, respectively, considered at the **left** and **right** part of the plot). Numbers in brackets represent the sum at the phylum level of relative abundances of all genera with significant change after AD calculated before and after AD (raw OW%/digestate%). Only genera with relative abundance ≥ 0.3% and significant change (-2 ≥ Log2 FC ≥ +2, *p*_*adj*_ < 0.05, minimum normalized read count = 10) are shown (see Supplementary Table 10 for the other groups).

**FIGURE 5:**
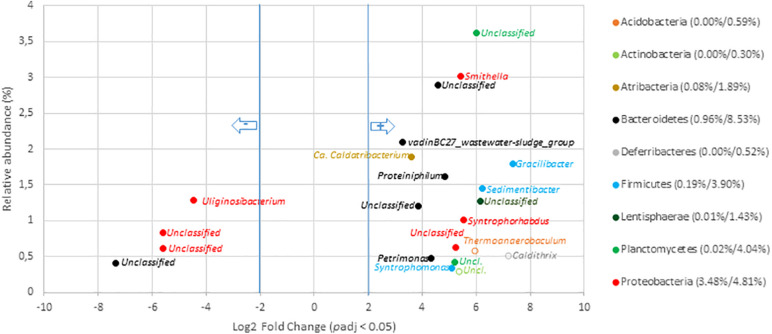
Genera with significant change in relative abundance after anaerobic digestion of raw organic wastes samples from urban origin. Genera with significant (*p*_*adj*_ < 0.05) decrease in digestates compared to raw OW samples (left part of the plot) are presented according to their initial relative abundance in raw OW. While genera with significant increase in digestates compared to raw OW samples (right part of the plot) are presented according to their relative abundance in digestate. Genera are colored according to the phylum to which they belong. Full mark represents genus present in the core microbiome while empty mark represents genus not present in the core microbiome (raw OW and digestate core microbiomes were, respectively, considered at the **left** and **right** part of the plot). Numbers in brackets represent the sum at the phylum level of relative abundances of all genera with significant change after AD calculated before and after AD (raw OW%/digestate%). Only genera with relative abundance ≥ 0.3% and significant change (-2 ≥ Log2 FC ≥ +2, *p*_*adj*_ < 0.05, minimum normalized read count = 10) are shown (see Supplementary Table 11 for the other groups).

To go deeper into the understanding of community changes, variations of V5–V6 16S rRNA gene reads allocated at the genus level were investigated. A DESEQ2 analysis was performed to highlight these changes (Figures 4, 5 and Supplementary Tables 10, 11). These analyses were performed with the non-resampled sequenced gene reads dataset (Supplementary Table 6) because a normalization scheme is directly run by DESEQ2. This approach revealed that the digestates of the central/agricultural category of samples led to a significant increase in the relative proportion of six genera: *Gelria* (*Firmicutes*), *Sporosarcina* (*Firmicutes*), *Dethiobacter* (*Firmicutes*), and three unclassified groups belonging to the *Spirochaetales*, *Cloacimonetes*, and *Firmicutes*. Four (*Gelria*, *Sporosarcina* and two others unclassified at genus level) of these genera were part of the core bacterial taxa found among digestates (Figure 4 and Supplementary Table 10). Four of these were also found to be part of the *Firmicutes* and confirmed the trends observed at the scale of phyla, which indicated an increase in *Firmicutes*. These six genera were two to five times (Log2 fold change) more abundant in the digestates than the non-treated samples (raw OWs). They represented from 0.1 to 6.1% (according to the mean normalized counts) of the total digestate bacterial community (Supplementary Table 10). Conversion of relative read numbers into 16S rRNA gene copies from the subsampled contingency table supported these trends and indicated an abundance of about 3.0 × 10^7^ *Gelria* 16S rRNA gene copies per gram of dry weight in the raw OW while 5.0 × 10^8^ *Gelria* 16S rRNA gene copies were estimated in average in the digestates (Supplementary Table 9). Similarly, *Sporosarcina* and *Dethiobacter* 16S rRNA gene copies per gram of dry weight indicated a gain of one log between the raw OW and digestates. Regarding genera that showed a relative decrease, 54 (of which 15 are parts of the core bacterial taxa found among digestates) (Supplementary Table 8) were subjected to a significant decrease after AD of OW of the agricultural/central sites (Figure 4 and Supplementary Table 10). This relative reduction varied from 2.6- to 9.9-fold when compared to raw OW (Figure 4 and Supplementary Table 10). *Lactobacillus* (4.8–0.1%), *Bacteroides* (4.1–0.4%), *Prevotella 9* (1.8–0.0%), and *Methanobrevibacter* (0.5–0.1%) were found to have undergone the most significant reductions (Figure 4 and Supplementary Table 10). The decrease in bacterial numbers was confirmed through a conversion of the dataset into 16S rRNA gene copies. *Lactobacillus* 16S rRNA gene copies went down by about two logs, and those of *Bacteroides* went down by about one log (supported by Wilcoxon rank sum tests; *p* < 0.05). Similar trends were observed for the other genera (Supplementary Table 9).

Several bacterial genera of the urban OWs were shown to be significantly enriched in the digestates. A total of 32 genera (of which 25 were part of the core microbiome) showed a significant increase (Figure 5 and Supplementary Table 11). These genera were enriched by three to sevenfold while comparing with the concentrations observed among OW. These genera represented from 0.03 to 3.6% of the total digestate microbial community. The most significant increases in the digestates were observed for V5–V6 16S rRNA gene reads allocated to the *Smithella* (0.03–3.0%), part of the *vadinBC27_wastewater-sludge_group* (0.1–2.1%), *Candidatus_Caldatribacterium* (0.1–1.9%), *Gracilibacter* (0.0–1.8%), *Proteiniphilum* (0.02–1.6%), *Sedimentibacter* (0.01–1.5%), *Syntrophorhabdus* (0.01–1.0%), and *Petrimonas* genus (0.01–0.5%). Conversion of these values into 16S rRNA gene copies showed the *Smithella* to have undergone an increase from 6.15 × 10^6^ copies per gram of OW to 2.64 × 10^9^ copies per gram of digestates, and the *Gracilibacter* 16S rRNA gene copies went up from about 3.0 × 10^6^ to 1.61 × 10^9^ (Supplementary Table 9). Similar trends were observed for the other above-cited genera (Supplementary Table 9) but a significant number (*n* = 18) of genera also underwent a relative reduction in V5–V6 gene reads from the OW to the digestates (Figure 5 and Supplementary Table 11). These decreases were about four- to ninefold in comparison to their relative numbers among the raw OW samples. Hence, the *Uliginosibacterium* (1.3–0.1%), *Simiduia* (0.3–0.0%), *Cellvibrio* (0.1–0.01%), *Fonticella* (0.1–0.0%) *Polyangium* (0.04–0.0%), and *Clostridium_sensu_stricto*_12 (0.03–0.0%) genera showed such a decrease (Figure 5 and Supplementary Table 11). These shifts were confirmed while converting these datasets into 16S rRNA gene copy numbers (Supplementary Table 9).

Global changes in the relative abundance of digestates’ bacterial genera significantly affected by ADs over the dataset were investigated. DESEQ2 analyses revealed that 30.8 and 35.5% of the total relative abundance of the digestate genera were significantly affected by the AD treatment, respectively, at the urban and agricultural/central sites (Supplementary Tables 10, 11). The relative abundances of 50 and 60 genera were significantly impacted by AD in, respectively, the urban and central/agricultural digestates. Despite this similar effect, a remarkable difference depending on OW origins was highlighted. In digestates of urban origin, this sorting mainly involved an enrichment of genera (32 out of the 50 impacted genera) while those produced from agricultural/central OW led essentially to a reduction of several taxa (54 out of 60 impacted genera) (Supplementary Tables 10, 11). These comparisons suggested a full re-shuffling of agricultural/central OW bacterial taxa while those of the urban OW appeared to be well adapted for the living conditions prevailing in the anaerobic digester.

#### Variations at the Scale of Genera Among Composts

**FIGURE 6:**
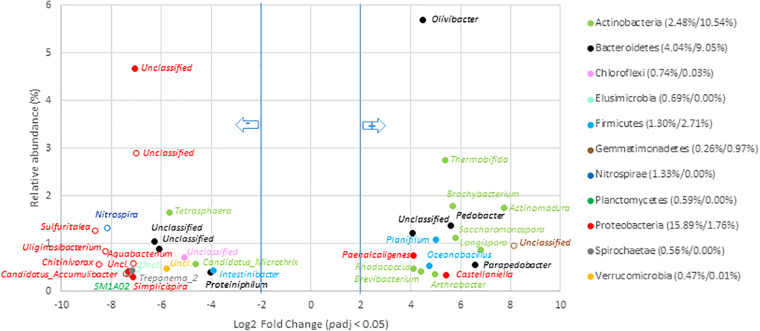
Genera with significant change in relative abundance after composting of organic waste samples. Genera with significant (*p*_*adj*_ < 0.05) decrease in compost compared to OW samples (left part of the plot) are presented according to their initial relative abundance in OW, while genera with a significant increase in compost compared to OW samples (right part of the plot) are presented according to their relative abundance in compost. Genera are colored according to the phylum to which they belong. Full mark represents genus present in the core microbiome; empty mark represents genus not present in the core microbiome (OW and compost core microbiomes were, respectively, considered at the left and right part of the plot). Numbers in brackets represent the sum at the phylum level of relative abundance of all genera with significant change after composting calculated before and after composting (OW%/Compost%). Only genera with relative abundance ≥ 0.3% and significant change (-2 ≥ Log2 FC ≥ +2, *p*_*adj*_ < 0.05, minimum normalized read count = 10) are shown (see Supplementary Table 12 for the other groups).

Composting was found to drive microbial communities toward similar V5–V6 genetic structures whatever the origin of the OW including those that had undergone AD (Figure 2A). These conserved compost microbiomes appeared to have been selected by strong environmental constraints that led to an increase in the relative abundance of, among others, *Actinobacteria*, *Deferribacteres*, *Firmicutes*, and *Thermotogae*. These trends were confirmed while considering the numbers of 16S rRNA gene copies between OW and composts for these phyla. In fact, a significant one log increase was observed for the *Actinobacteria* and *Firmicutes* per gram of composts (Supplementary Table 9). Furthermore, the 16S rRNA gene copies allocated to the *Thermotogae* and *Deferribacteres* went from a negligible score up to several millions. Simultaneously, V5–V6 reads of six phyla (*Nitrospirae*, *Elusimicrobia*, *Acidobacteria*, *Saccharibacteria*, TM6, and GOUTA4) became negligible and were associated to a two-log reduction in 16S rRNA gene copies (Supplementary Table 9). These shifts among these phyla were confirmed at the scale of genera (Figure 6 and Supplementary Table 12). The compost microbiome was enriched in 36 genera of which only 6 were versatile and not found among all composts (Figure 6 and Supplementary Table 12). Relative distribution patterns of these genera changed by 4.1- to 8.1-Log2 fold when compared to the non-treated OW samples (going from 0.04 to 5.7% of the total compost microbial community). The most significant relative distribution increases were observed for reads allocated to the *Actinomadura*, *Longispora*, *Parapedobacter*, *Saccharomonospora*, *Brachybacterium*, *Pedobacter*, *Thermobifida*, *Planifilum*, *Oceanobacillus*, *Olivibacter*, and *Paenalcaligenes*. These genera are part of the *Actinobacteria* (50%), *Bacteroidetes*, or *Firmicutes*. In most cases, about two log increases in 16S rRNA gene copy numbers were associated with these changes (all statistical tests were significant; Wilcoxon *p* < 0.05) (Supplementary Table 9). For example, the number of 16S rRNA copies for the *Olivibacter* went up from 2.0 × 10^8^ to 1.0 × 10^10^. Most impressive was the shift from negligible numbers of *Longispora* among the OW going up to about 2.0 × 10^9^ 16S rRNA gene copies (Supplementary Table 9). These changes were associated with the relative decreases of V5–V6 16S rRNA gene reads allocated to 105 genera including 29 found in the raw OWs core microbiome (Figure 6 and Supplementary Table 12). This reduction went from 3.8- to 8.1-Log2 fold change compared to OW where these genera originally represented 0.02–4.7% of the total raw OW microbial community (Figure 6 and Supplementary Table 12). Reads from *Tetrasphaera* (1.7–0.04%), *Microthrix* (0.6–0.03%), *Intestinibacter*_9 (0.4–0.04%), *Treponema*_2 (0.4% to 0.0%), *Proteiniphilum* (0.4–0.04%), *Simplicispira* (0.3–0.0%), *Christensenellaceae*_R7_group (0.3–0.03%), *Lautropia* (0.2–0.0%), *Macellibacteroides* (0.2–0.01%), BD17_clade (0.1–0.0%), *Propionivibrio* (0.1–0.0%), *Proteocatella* (0.1–0.0%), and *Streptococcus* (0.1–0.0%) in the OW were the most significantly impacted (Figure 6 and Supplementary Table 12). These decreases among the community were confirmed by an analysis of the equivalent 16S rRNA gene copies for these genera in the OW and composts (Supplementary Table 9). DESEQ2 analyses indicated that a re-shuffling of 52.6% of the read distribution patterns occurred during composting of OW. Composting was thus highly selective with a decline of 28.3% of the total relative abundance of the compost’s bacterial taxa and an enrichment of 24.4% of the other taxa in the composts (Supplementary Table 12).

### Incidence of OW Digestion Processes on Bacterial Functional Groups

Functional traits selected by AD and composting were inferred from the taxonomic allocations performed at the genus level. At first, the sum of relative abundances of enriched genera potentially covering a functional trait extracted from the FAPROTAX database using MACADAM was calculated (Supplementary Table 13). The contribution of each genus to differences in hypothetical functional profiles is based on relative abundances before and after treatment (Supplementary Table 13). Based on the most abundant taxa in digestates from urban origin, the five main hierarchical functional traits were chemoheterotrophy (6.3%), fermentation (6.2%), human and mammal gut colonizers (each feature at 2.1%), and animal parasites or symbionts (2.1%) (Supplementary Table 13D). Similarly, these functional traits were also associated with the most abundant taxa in digestates from agricultural and central origin (Supplementary Table 13F). In composts, the functional traits of the enriched genera (as suggested from the number of equivalent 16S rRNA gene copies) were related to aliphatic non-methane hydrocarbon degradation, arsenate detoxification, dissimilatory arsenate reduction, hydrocarbon degradation, nitrate and nitrite ammonification, nitrate and nitrite respiration, and oil bioremediation. These taxa had increased by at least 8.5 log after composting of raw OWs (Supplementary Table 13B).

Functional traits related to health hazards were also investigated based on changes in relative abundances of bacterial genera before and after OW treatment (Supplementary Table 13). AD and composting processes contributed significantly at reducing genera potentially harboring infectious agents. The number of 16S rRNA gene copies of genera potentially causing diseases in human, plants, or fishes decreased by 9.3, 7.4, and 7.9 log, respectively, after composting of OW (Supplementary Table 13A). Similar trends could be observed during AD for genera harboring potential human pathogens. The number of 16S rRNA gene copies of genera potentially associated with human pathogens went down by about 7.4 and 1.7 log, respectively, in digestates from urban and agricultural/central categories (Supplementary Tables 13C,E). Interestingly, the number of 16S rRNA gene copies of genera possibly including lung pathogens or plant pathogens went down by about 7.5 log in digestates from the agricultural/central category (Supplementary Table 13E).

The theoretical metabolic pathways of the genera identified in this study were further inferred from sequenced genomes using the MACADAM software. Metabolic pathways (with a PS ≥ 0.5) potentially present in genera significantly impacted by the investigated digestion processes were counted, and their relative distribution patterns were compared (Table 2 and Supplementary Table 14). Distribution patterns between these functional categories observed among OWs from urban sites and from central/agricultural ones were found positively correlated (Spearman Rank Correlation; rho = 0.69, *p* < 0.05). Such correlations were not observed between the anaerobic and aerobic digestion processes (digestates and composts).

**TABLE 2:** Metabolic pathways recorded among the bacterial genera significantly impacted by the anaerobic digestion or composting processes as observed by the MACADAM approach.

Main hierarchical groups of pathways			Percentage of theoretical metabolic pathways^*a*^ based on available sequenced genomes of isolates correlated to significantly impacted genera^*b*^ in								
	
			Digestates from urban origin			Digestates from agricultural/central origin			Compost		
			
			TMP-EG (*n* = 252)	TMP-DG (*n* = 645)	*R*	TMP-EG (*n* = 321)	TMP-DG (*n* = 746)	*R*	TMP-EG (*n* = 753)	TMP-DG (*n* = 800)	*R*
Biosynthesis pathways	Cofactor^*c*^		23.0	15.0	1.5	21.8	11.7	1.9	13.8	13.5	1.0
	Amino acid		13.9	8.7	1.6	12.8	6.4	2.0	8.6	8.6	1.0
	Nucleotide		9.9	6.1	1.6	8.4	3.6	2.3	5.1	5.3	1.0
	Lipid		7.5	4.3	1.7	4.7	4.0	1.2	4.3	4.5	1.0
	Carbohydrates		4.8	5.1	0.9	2.2	4.8	0.5	4.5	5.4	0.8
	Aminoacyl-tRNAs-charging		1.2	0.5	2.4	0.9	0.1	9.0	0.4	0.4	1.0
	Secondary metabolite		1.2	2.2	0.5	0.9	3.0	0.3	2.7	2.4	1.1
	Cell structure		0.8	1.6	0.5	0.6	1.3	0.5	1.1	1.8	0.6
	Metabolic regulators		0.8	1.4	0.6	0.3	1.6	0.2	1.7	1.6	1.1
	Aromatic compounds		0.8	1.1	0.7	1.3	1.1	1.2	1.3	1.1	1.2
	Alcohol		0.4	1.7	0.2	0.6	1.3	0.5	1.2	1.3	0.9
	Hormone		0.0	0.6	0	0.0	0.4	0	0.5	0.5	1.0
Degradation pathways	Amino acid		6.4	7.4	0.9	8.1	8.3	0.9	7.6	7.8	1.0
	Nucleotide		4.8	3.6	1.3	4.4	2.7	1.6	2.8	3.0	0.9
	Fatty acid and lipid		0.0	1.4	0	0.6	1.2	0.5	1.2	1.4	0.9
	Carbohydrates		4.8	7.1	0.7	2.5	7.6	0.3	5.4	6.4	0.8
	Secondary metabolite		2.0	3.3	0.6	1.6	4.2	0.4	4.1	3.3	1.2
	Aromatic compounds		0.0	2.0	0	0.9	7.4	0.1	7.7	5.3	1.5
	Alcohol		1.2	1.7	0.7	1.3	1.3	1.0	1.3	1.5	0.9
	Carboxylates		3.2	5.0	0.6	4.1	5.6	0.7	5.1	5.1	1.0
	C1 compounds^*d*^		2.0	2.3	0.9	2.8	2.7	1.0	2.1	2.4	0.9
	Non-carbon nutrients	Sulfur metabolism	1.2	2.3	0.5	2.2	4.0	0.6	2.4	2.9	0.8
		Phosphorus compounds	0.4	0.8	0.5	1.3	1.2	1.1	1.2	1.0	1.2
Energy-metabolism	Anaerobic respiration		1.6	1.9	0.8	1.9	3.2	0.6	2.8	2.9	1.0
	Aerobic respiration		1.2	1.6	0.8	2.8	1.6	1.8	1.6	1.6	1.0
	Fermentation		1.2	4.7	0.3	3.1	3.5	0.9	3.3	3.3	1.0
	Hydrogen-production		1.2	0.8	1.5	0.6	0.7	0.9	0.7	0.6	1.2
	Hydrogen-oxidation		0.4	0.5	0.8	0.3	0.4	0.8	0.4	0.4	1.0
Others	Nitrogen cycling processes^*e*^		1.6	3.1	0.5	4.1	2.7	1.5	2.8	2.8	1.0
	Detoxification^*f*^		1.2	1.9	0.6	2.2	2.0	1.1	1.9	1.9	1.0
	Nucleic acid processing		1.6	0.6	2.7	0.9	0.3	3.0	0.5	0.5	1.0

*^a^A minimum pathway score (PS) and pathway frequency score (PSF) of 0.5 was used for hypothetical metabolic pathways filtering.*

*^b^To see the list of organisms with the targeted metabolic pathway, see Supplementary Tables 14A–F. ^*c*^Cofactor biosynthesis are cobyrinate-diamide biosynthesis, vitamin biosynthesis, CoA biosynthesis, reductants, NAD metabolism, porphyrin compounds biosynthesis, quinone biosynthesis, polyprenyl biosynthesis, molybdenum cofactor, lipoate biosynthesis, and tetrapyrrole biosynthesis. ^*d*^C1 compounds are CO_2_ fixation, formaldehyde oxidation, methanol oxidation, formaldehyde assimilation, and methane oxidation. ^*e*^Nitrogen cycling processes consist of nitrate reduction, ammonia assimilation, ammonia oxidation, denitrification, taurine degradation, and nitrogen fixation. ^*f*^Detoxification involves acid resistance, arsenate detoxification, methylglyoxal detoxification, antibiotic resistance, and reactive oxygen species degradation. Abbreviations: TMP-EG, theoretical metabolic pathways in the enriched genera after treatments; TMP-DG, theoretical metabolic pathways in the decaying genera; R, relative proportion of pathways per category between the enriched genera and the decaying ones.*

After AD treatments, biosynthesis pathways for cofactors, nucleotides, amino acids, lipids, and aminoacyl-tRNAs charging were more abundant in the enriched genera than in the decaying ones whatever the raw OW origin (Table 2 and Supplementary Table 14). Whereas biosynthesis pathways for secondary metabolites, cell structure, metabolic regulators, and alcohol and hormone biosynthesis were less abundant in the enriched genera than in the decaying ones (Table 2 and Supplementary Table 14). Similarly, degradation pathways for fatty acids and lipids, carbohydrates, secondary metabolites, aromatic compounds, and sulfur-containing compounds metabolism degradation were less abundant in the enriched genera than the decaying ones (Table 2 and Supplementary Table 14). Interestingly, in digestates from agricultural and central origin, nitrogen cycle-related processes (nitrate-reduction, ammonia-assimilation, ammonia-oxidation, denitrification, taurine-degradation, and nitrogen-fixation pathways) were more abundant in the enriched genera than the decaying ones, but the opposite situation occurred among digestates of urban origin (Table 2 and Supplementary Table 14). After composting treatment, excepting the biosynthesis pathways involved in cell structure and the aromatic compounds degradation pathways, the inferred pathways (*n* = 29) had a similar distribution among the impacted genera (Table 2 and Supplementary Table 14). Cell structure biosynthesis pathways were less abundant in the enriched genera after both types of treatment (AD and composting) than in the decaying ones. Aromatic compounds degradation pathways were more abundant in the enriched genera found among composts but not among those of digestates (Table 2 and Supplementary Table 14).

## Discussion

OW recycling of sewage sludge and livestock effluents represent a major challenge because not only the bioenergy production but also environmental and sanitary issues must be considered. A key criterion in the assessment of the microbiological risks of OW recycling that remain poorly defined is the nature and structure of the various microbiomes getting into these processes and their potential health and ecological hazards. Here, the core and flexible components of the end product microbiomes were inferred, and the likelihood of these at being reproducibly found among highly diverse OW treatment plants was evaluated. Nine full-scale treatment plants distributed over France were selected to obtain a global view of microbiome bacterial taxa in the French OW treatment sector. These inferred bacterial taxa (from metabarcoding 16S rRNA gene datasets) are discussed below according to process type (AD, composting) and OW origins (fecal wastes from WWTP or agricultural sites, and food products). Key functional traits of the bacterial genera impacted by the digestion processes were investigated to go further with the identification of key structuring bacterial processes.

According to NMDS ordinations of 16S rRNA gene metabarcoding datasets, two main bacterial community structures could be identified and found to match a segregation between bacterial taxa from OW containing agricultural wastes or not (restricted to urban WWTP activated sludge). This segregation was also in line with a differentiation between their contents in micropollutants particularly the TEs. The urban samples (treated or not) had significantly more micropollutants including 14 TEs (Ag, Al, As, Cd, Ce, Co, Cr, Cu, Ga, Mo, Ni, P, Pb, and Ti), 4-nonylphenol, and 2 PAHs (phenanthrene and anthracene) than the agricultural and mixed (agricultural/urban; termed “central” in the text) samples. The 16S rRNA gene microbial community structures of the investigated OW urban samples were in agreement with previous studies (Juretschko et al., 2002; Wong et al., 2005; Hu et al., 2012; Díaz et al., 2018). Inferred 16S rRNA gene taxonomic allocations were predominantly among the *Proteobacteria*, *Bacteroidetes*, *Actinobacteria*, *Firmicutes*, and *Nitrospirae* phyla. *Proteobacteria* were reported to be recurrent among WWTP activated sludge and have been shown to represent almost 50% of the bacterial taxa (Juretschko et al., 2002; Wong et al., 2005; Hu et al., 2012). *Actinobacteria* were also previously mentioned as highly abundant organisms in such wastes and being favored by the high phosphorus content of activated sludge (Wagner et al., 1994). *Nitrospirae* known to be nitrite-oxidizing bacteria were previously considered as common in activated sludge but were not mentioned as being abundant core organisms (Saunders et al., 2016).

After AD of both categories of raw OWs (urban and central/agricultural categories), 16S rRNA gene inferred phyla with similar relative changes were observed. These shifts occurred whatever the raw OW origin. Both categories of digestates (urban and central/agricultural categories) were impacted by selective environmental changes, which led to a decrease in the relative abundance of *Proteobacteria* but an enrichment in *Firmicutes* (which nearly doubled in relative abundance). *Firmicutes* had been reported by several authors as one of the most abundant phyla in AD processes of activated sludge (Hu et al., 2012; Zhang et al., 2012; Abendroth et al., 2015; Díaz et al., 2018). Sundberg et al. (2013) also found that *Firmicutes* were the most prevalent in samples taken from reactors fed with various combinations of wastes (from slaughterhouses, restaurants, households, etc.). In this work, with 52.2% of V5–V6 rRNA gene reads, *Firmicutes* were dominant in the digestates from central (mixed)/agricultural sites. Furthermore, they were enriched by 2.6-fold in digestates from urban sites to reach an 18.4% relative abundance. These results seem to indicate that the AD conditions favored their growth whatever the OW origins. To go deeper into the understanding of these community changes, the impact of AD on the structuration of end product microbiomes was assessed by grouping 16S rRNA gene reads according to their allocations at the genus level, and making comparisons by DESEQ2. These analyses revealed that about one third of the total relative abundance (according to the mean normalized counts) of the digestate bacterial taxa were significantly affected by the AD treatment whatever the raw OW origin. The relative abundances of 50 and 60 genera were significantly impacted by AD as observed from, respectively, urban and central/agricultural digestates. However, differences in the shifts of the 16S rRNA gene reads of these taxa were found related to the origin of the OW. In digestates of urban origin, this sorting mainly implied an increase in the relative abundance of OW urban taxa while those generated from agricultural/central OW led essentially to a reduction in the relative abundance of most of the initial OW taxa. This indicates that the coalescence of bacterial taxa of raw OW with the microbial community of the digesters will be largely dependent on their origin (and nature). In fact, urban OWs (mainly composed of activated sludge) were found to contain better fit communities for the AD process that could interact with the digesters’ microbial communities. Bacterial taxa present in agricultural raw OW were outcompeted by the digesters’ communities. This could be explained by the pre-treatment conditions performed in municipal wastewater facilities, leading to an increase of specialized bacterial taxa in the activated sludge. These taxa can perform multiple biodegradation processes (Shchegolkova et al., 2016) and can form heterogeneous structures termed flocs (Ju and Zhang, 2015). Functional inferences through FAPROTAX (Louca et al., 2016) were undertaken to highlight their potential contributions to the AD process.

The functional traits of the dominant microbial taxa inferred from the 16S rRNA gene datasets in the AD end products were investigated. Based on FAPROTAX analyses, functional traits such as chemoheterotrophy and fermentation were highly common among the taxa found in digestates of the urban and agricultural/central categories. Chemoheterotrophic bacteria are usually acting as primary decomposers (hydrolysis step), which are responsible for OW recycling in diverse ecosystems (Kämpfer et al., 1993). Fermentation, a widespread anaerobic pathway, is an essential step in AD process where fermentative bacteria convert simple monomers such as simple sugar, amino acids, and alcohols into short-chain fatty acids, primary alcohols, as well as H_2_ and CO_2_ (Cardinali-Rezende et al., 2009; Bajpai, 2017; Anukam et al., 2019). Interestingly, these functions (chemoheterotrophy and fermentation) were also previously reported by Wei et al. (2018) as the most dominant functions in activated sludge. This elucidates why bacterial genera from activated sludge could directly contribute to the observed diversity in the digestates. This observation is in line with the hypothesis of a selection of the most fit K-strategists (specialists) with ecologically relevant functions during the AD process. Also, these results confirmed that the changes in the AD community composition were driven by functional traits and less constrained by taxonomic relatedness.

To corroborate the above conclusion, closer analyses of the metabolic potential of bacterial communities were performed. Pathways inferred to be present in the enriched genera after treatment against those of the decreased ones were compared according to the origin/natures of the OWs. Interestingly, AD treatment was confirmed to impact similarly numerous metabolic pathways in both categories of OWs (urban and central/agricultural categories). Ten biosynthesis pathways (10/12) involved in the production of secondary metabolites, cell structure, metabolic regulators, biosynthesis, etc., and nine degradation pathways (9/11) (e.g., aromatic compounds and sulfur-containing compounds degradation) were affected similarly in both digestates from urban and agricultural/central categories. Nevertheless, some categories of pathways were not impacted in the same way according to the OW origin. For example, in digestates from agricultural and central origin, nitrogen cycling processes (e.g., nitrate-reduction, ammonia-assimilation, denitrification, and nitrogen-fixation pathways) were more abundant in the enriched genera than in the decaying ones, but the opposite situation was observed for digestates of urban origin. These pathways (related to nitrogen cycle) have been reported by Wei et al. (2018) as central key functions in activated sludge. Thus, these pathways could be critical in the ecology of activated sludge but less important during AD.

The impact of composting per OW type on the bacterial communities was also investigated. After composting, a NMDS plot grouped together the 16S rRNA community profiles whatever the OW origin. Composting was found to drive bacterial communities toward similar genetic structures in all samples without differentiating OW inputs that had previously undergone AD. This conserved microbiome appeared to have been selected by highly selective constraints leading to a significant decrease in the relative abundance of *Bacteroidetes*, *Chlorobi*, *Chloroflexi*, *Lentisphaerae*, *Planctomycetes*, *Proteobacteria*, *Verrucomicrobia*, and *Spirochaetae* and the loss of six phyla (*Nitrospirae*, *Elusimicrobia*, *Acidobacteria*, *Saccharibacteria*, *TM6*, and *GOUTA4*) from the inputs OW. Other studies reported similar findings (Steger et al., 2007a; Song et al., 2014). In a compost pile, changes in physicochemical factors such as pH, temperature, moisture, and organic carbon were found to be correlated with bacterial growth. Microbial community structures were found to be affected by these evolving parameters (Cahyani et al., 2003; Liang et al., 2003; Tang et al., 2007; Zhang et al., 2011). Among these, temperature was shown to be highly selective (Palmisano and Barlaz, 1996; Steger et al., 2007a). Interestingly, with their large shift in relative abundance (4.3-fold) observed between OW and composts, *Actinobacteria* showed a major re-shuffling with an up to 20% increase. These are clearly key players in the composting process.

To go deeper into the understanding of these effects, changes in the composition of the compost bacterial taxa were investigated at genus level. A total of 141 genera were found to be affected by composting, with 105 genera showing a significant decrease (equivalent to 53.7% of the total relative abundance of the compost’s bacterial taxa). Only three of these decaying genera were allocated to the *Actinobacteria.* However, 16 of the 36 enriched genera were part of this phylum. Previous studies have reported the persistence of *Actinobacteria* over the composting process from the thermophilic to the curing stage, regardless of the nature of treated OW (Danon et al., 2008). This bacterial group also constituted a significant part of the microbial community in the late composting stages (Herrmann and Shann, 1997; Tiquia et al., 2002; Hiraishi et al., 2003; Steger et al., 2007b). *Actinobacteria* are Gram-positive bacteria with high G + C contents in DNA, widely distributed in a diversity of ecological environments, including extreme ecosystem (Qin et al., 2011, 2019; Dhakal et al., 2017; Goodfellow et al., 2018). They can perform several important functions. Some *Actinobacteria* can synthesize a wide diversity of secondary metabolites, which may promote survival in highly selective environments (Fang et al., 2017; Puttaswamygowda et al., 2019; Qin et al., 2019). Several studies have highlighted the importance of temperature as a selective factor of thermo−tolerant *Actinobacteria*. In fact, *Saccharomonospora viridis*, *Thermobifida fusca*, and *Thermobispora bispora* have been reported in the later composting stages. In our study, many genera of thermo−tolerant *Actinobacteria*, e.g., *Thermobifida*, *Actinomadura*, *Saccharomonospora*, *Arthrobacter*, *Thermomonoospora*, and *Thermocrispum* were enriched in compost irrespective of the origin of the treated OW. *Actinobacteria* are capable of breaking down a wide range of animal wastes and plant debris particularly complex compounds (e.g., lignocelluloses), playing an important role in the later stages of composting (Herrmann and Shann, 1997; Wang et al., 2016). It is to be noted that thanks to the occurrences of bacterial genera degrading xenobiotics, composting offers the advantage of decreasing the concentration of chemical contaminants (Semple et al., 2001; Ghaly., 2011; Cascant et al., 2016; Ren et al., 2018). In line with these reports, genera of xenobiotic degrading *Actinobacteria*, e.g., *Arthrobacter*, were found to be enriched in the 16S rRNA gene datasets generated from composts DNA. To go deeper, closer analyses of functional potentials *per* taxa enriched after composting were performed. The functional profiles of the enriched genera suggested that the number of 16S rRNA copies of genera, e.g., *Arthrobacter*, *Alcanivorax*, *Rhodococcus*, *Brevibacterium*, etc., known to consist of many strains having functional traits involved in arsenate detoxification, dissimilatory arsenate reduction, plastic degradation, and oil bioremediation have significantly increased through composting. This further supports the idea that composting is an efficient bioremediation process.

## Conclusion

In conclusion, AD and composting induced bacterial changes that were found driven by pH, temperature, but also the C- and energy sources which require specialized metabolic properties. These factors were more selective in composting than AD. Consequently, composting generated highly predictable community patterns whatever the OW origin. In AD, the relative contribution of the raw OW microbiomes on the digestate structural and functional bacterial community patterns was dependent on its origin. Bacterial genera from activated sludge were found to be well-suited for the AD process and contributed directly to the observed diversity observed in the digestates. Content in activated sludge among the OW was thus considered to be a critical factor for predicting the digestate bacterial contents. Our findings provided new insights into our understanding of bacterial coalescence phenomena in aerobic and anaerobic digesters. However, knowledge gap remains regarding potential health hazards associated with the emerging communities observed in the end products. These organic matter biodegradation processes being built from a large panel of exogenous communities, they might offer conditions favorable for horizontal gene transfer (HGT) events such as the HGT of antibiotic resistance genes (ARG). The next step in these analyses will be to evaluate the frequency of these HGT.

## Data Availability Statement

The datasets presented in this study can be found in online repositories. The names of the repository/repositories and accession number(s) can be found below: European Nucleotide Archive (https://www.ebi.ac.uk/research) under the accession number PRJEB40193.

## Author Contributions

AA, EB, LM, BC, and WG conceived and designed experiments. AA, EB, LM, SH, DP, ED, BC, and WG performed the experiments. AA, EB, BC, and WG performed the data curation and statistical analyses. AA, BC, and WG wrote the manuscript. All authors read and approved the final manuscript.

## Conflict of Interest

The authors declare that the research was conducted in the absence of any commercial or financial relationships that could be construed as a potential conflict of interest.

## Footnotes

1 https://www.arb-silva.de/documentation/
