# Supplementary material for: Relative Weight of Organic Waste Origin on Compost and Digestate 16S rRNA Gene Bacterial Profilings and Related Functional Inferences
Source: Front Microbiol. 2021 May 14;12:667043. doi: 10.3389/fmicb.2021.667043 (PMC8160089; doi:10.3389/fmicb.2021.667043)
Supplement: Supplementary file 1 [file Presentation_1.PPTX]

## Slide 1
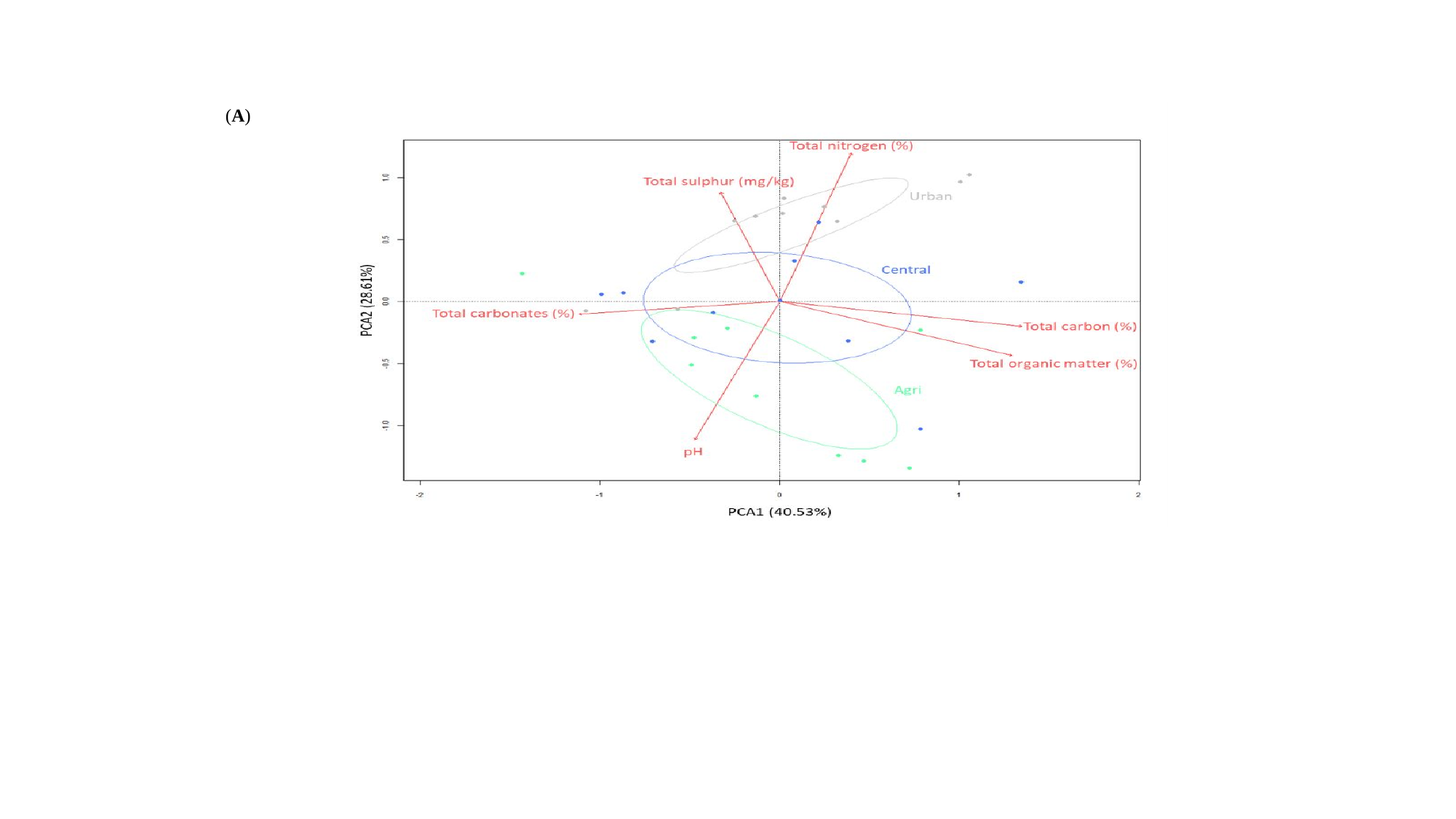

(A)

## Slide 2
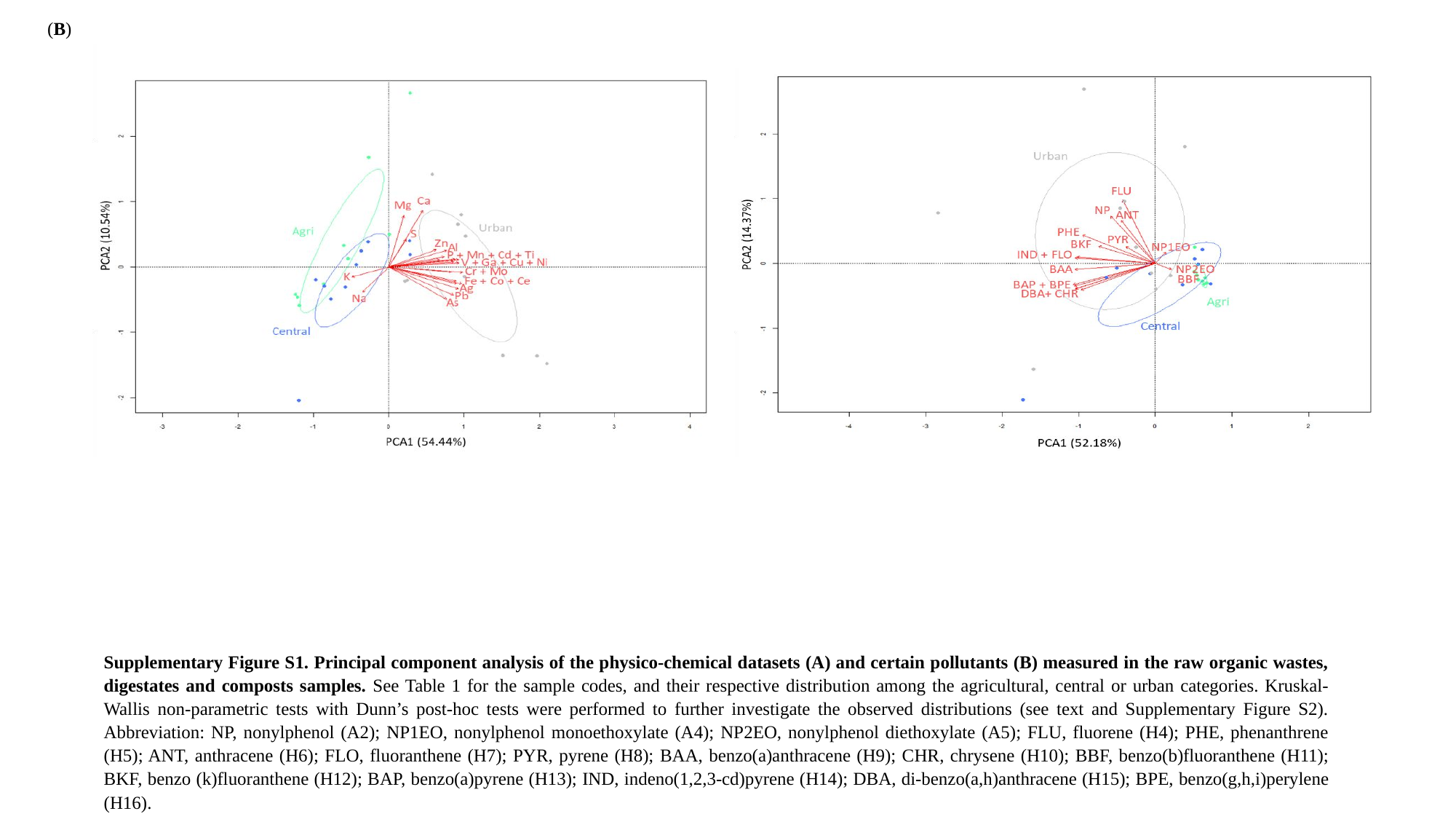

(B)
Supplementary Figure S1. Principal component analysis of the physico-chemical datasets (A) and certain pollutants (B) measured in the raw organic wastes, digestates and composts samples. See Table 1 for the sample codes, and their respective distribution among the agricultural, central or urban categories. Kruskal-Wallis non-parametric tests with Dunn’s post-hoc tests were performed to further investigate the observed distributions (see text and Supplementary Figure S2). Abbreviation: NP, nonylphenol (A2); NP1EO, nonylphenol monoethoxylate (A4); NP2EO, nonylphenol diethoxylate (A5); FLU, fluorene (H4); PHE, phenanthrene (H5); ANT, anthracene (H6); FLO, fluoranthene (H7); PYR, pyrene (H8); BAA, benzo(a)anthracene (H9); CHR, chrysene (H10); BBF, benzo(b)fluoranthene (H11); BKF, benzo (k)fluoranthene (H12); BAP, benzo(a)pyrene (H13); IND, indeno(1,2,3-cd)pyrene (H14); DBA, di-benzo(a,h)anthracene (H15); BPE, benzo(g,h,i)perylene (H16).

## Slide 3
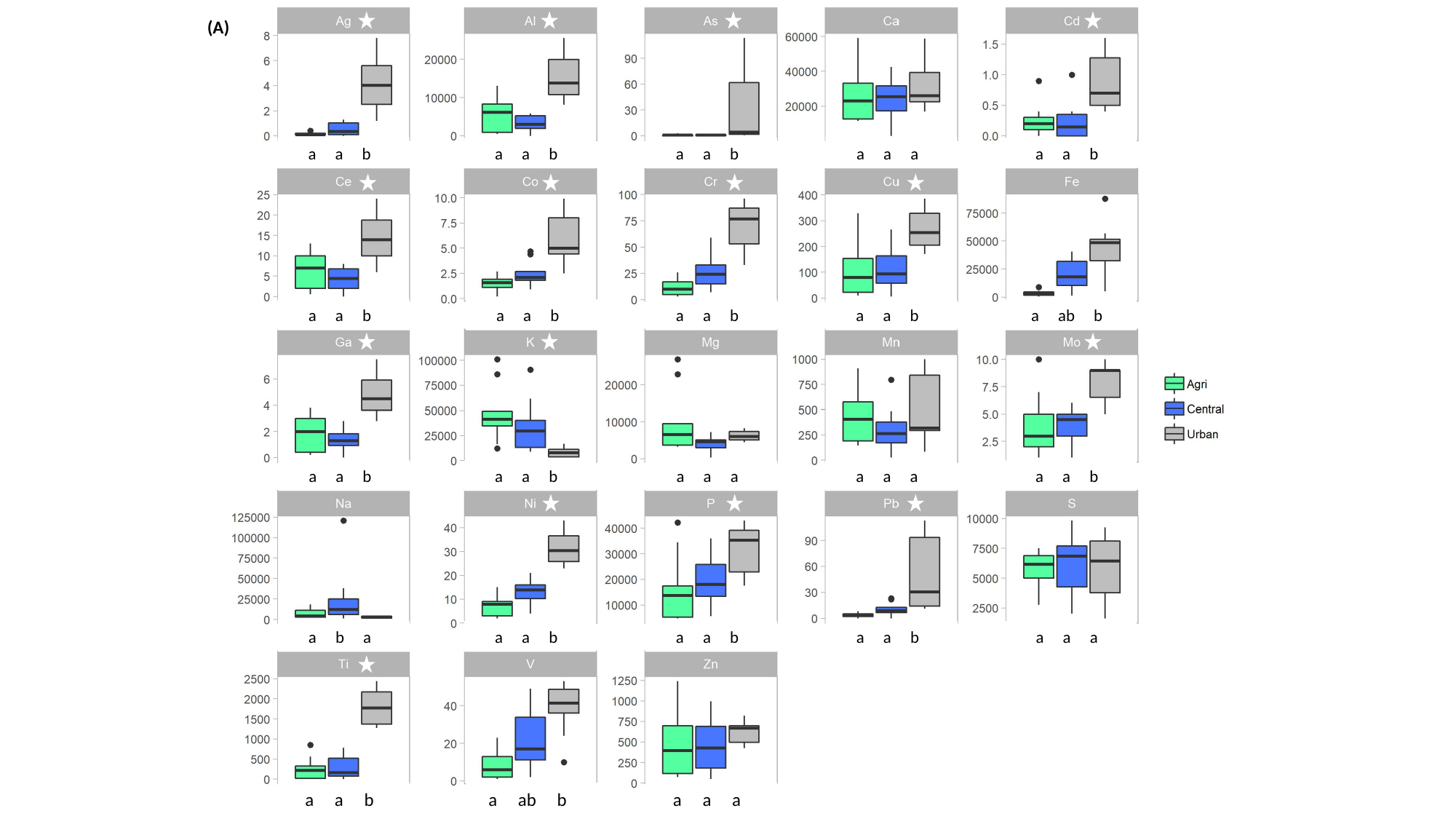

(A)
a a b
a a b
a a b
a a a
a a b
a a b
a a b
a a b
a a b
a ab b
a a b
a a b
a a a
a a a
a a b
a b a
a a b
a a b
a a b
a a a
a a b
a ab b
a a a

## Slide 4
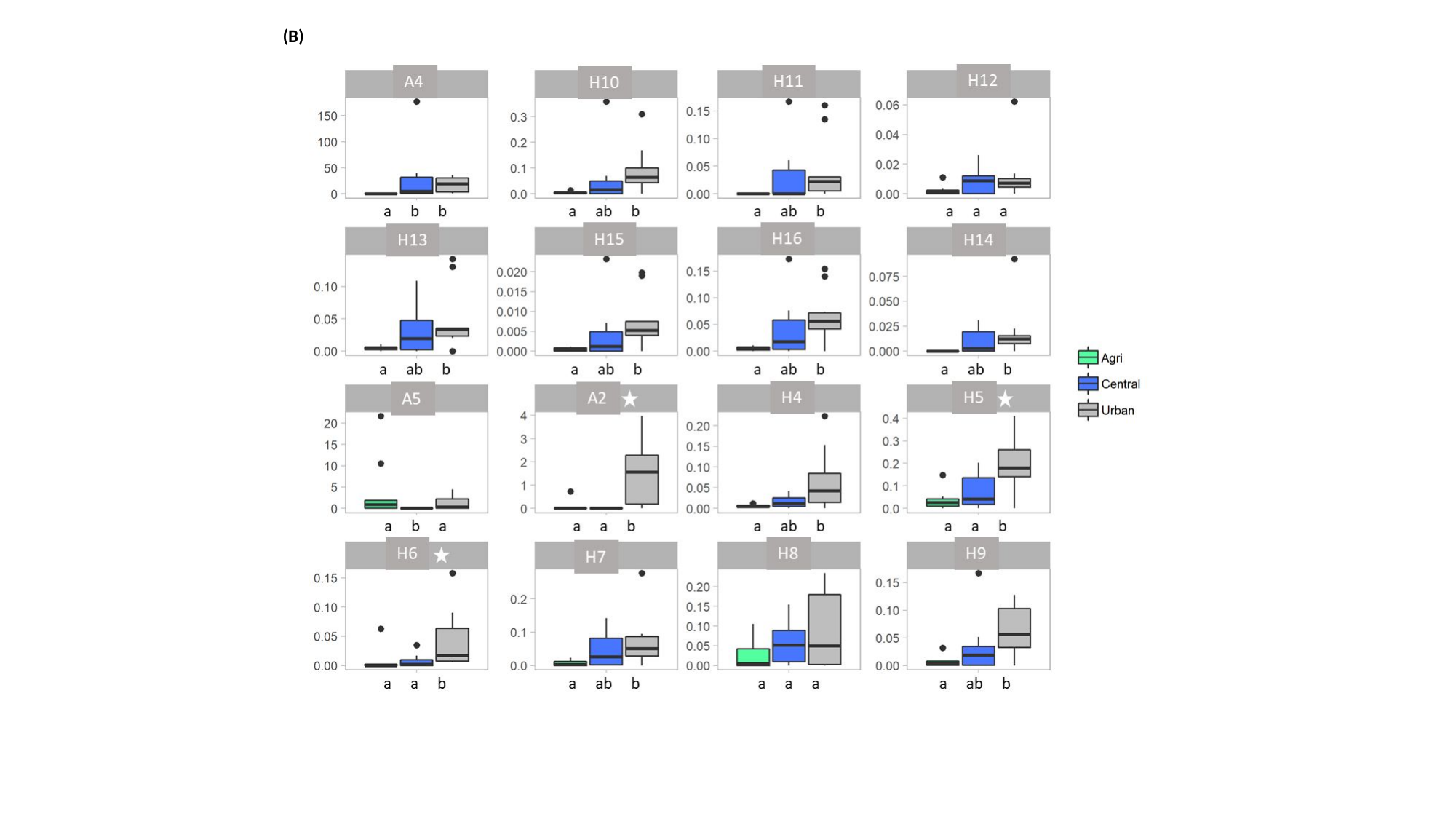

(B)

## Slide 5
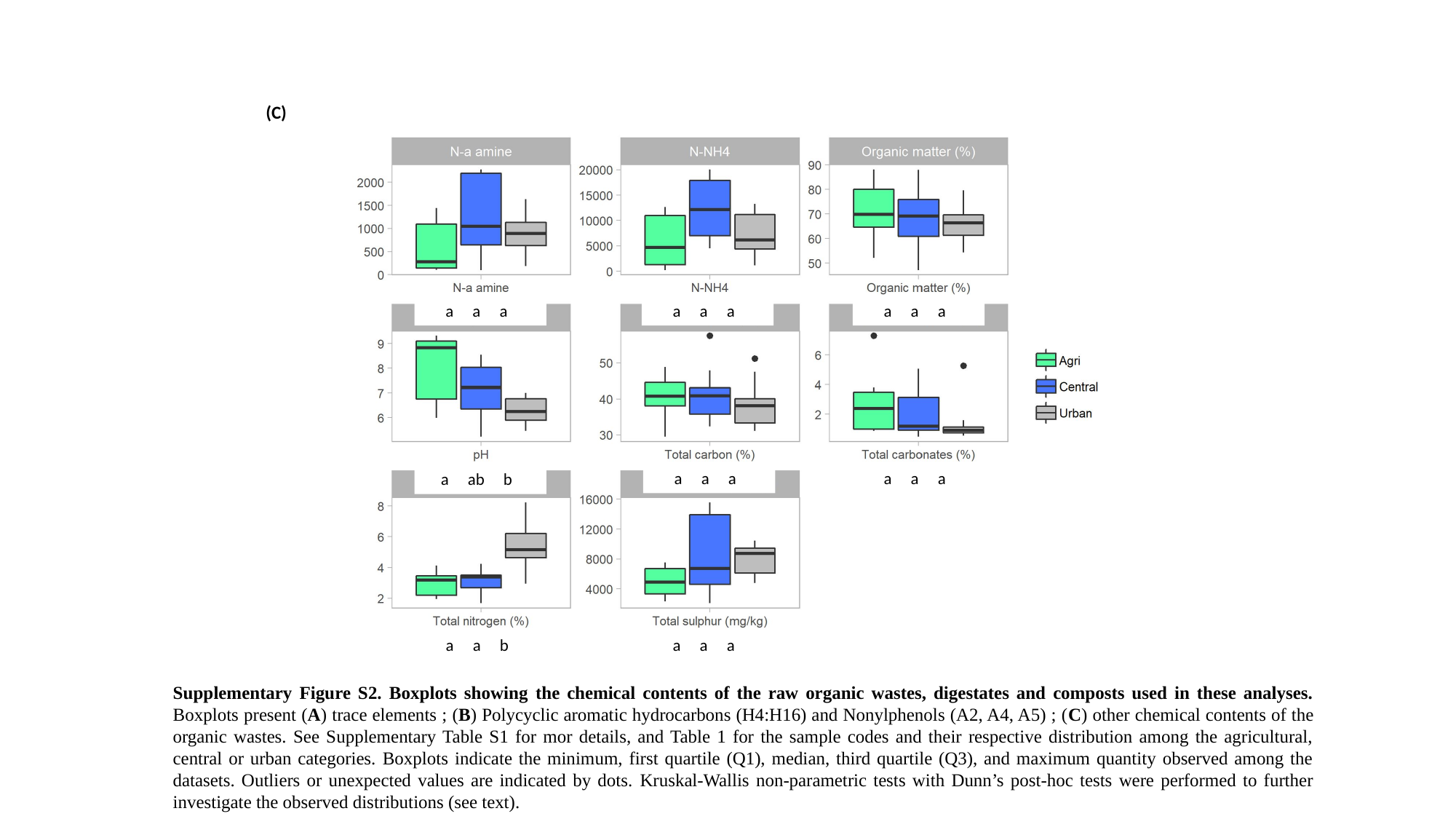

(C)
a a a
a a a
a a a
a a a
a a a
a ab b
a a b
a a a
Supplementary Figure S2. Boxplots showing the chemical contents of the raw organic wastes, digestates and composts used in these analyses. Boxplots present (A) trace elements ; (B) Polycyclic aromatic hydrocarbons (H4:H16) and Nonylphenols (A2, A4, A5) ; (C) other chemical contents of the organic wastes. See Supplementary Table S1 for mor details, and Table 1 for the sample codes and their respective distribution among the agricultural, central or urban categories. Boxplots indicate the minimum, first quartile (Q1), median, third quartile (Q3), and maximum quantity observed among the datasets. Outliers or unexpected values are indicated by dots. Kruskal-Wallis non-parametric tests with Dunn’s post-hoc tests were performed to further investigate the observed distributions (see text).

## Slide 6
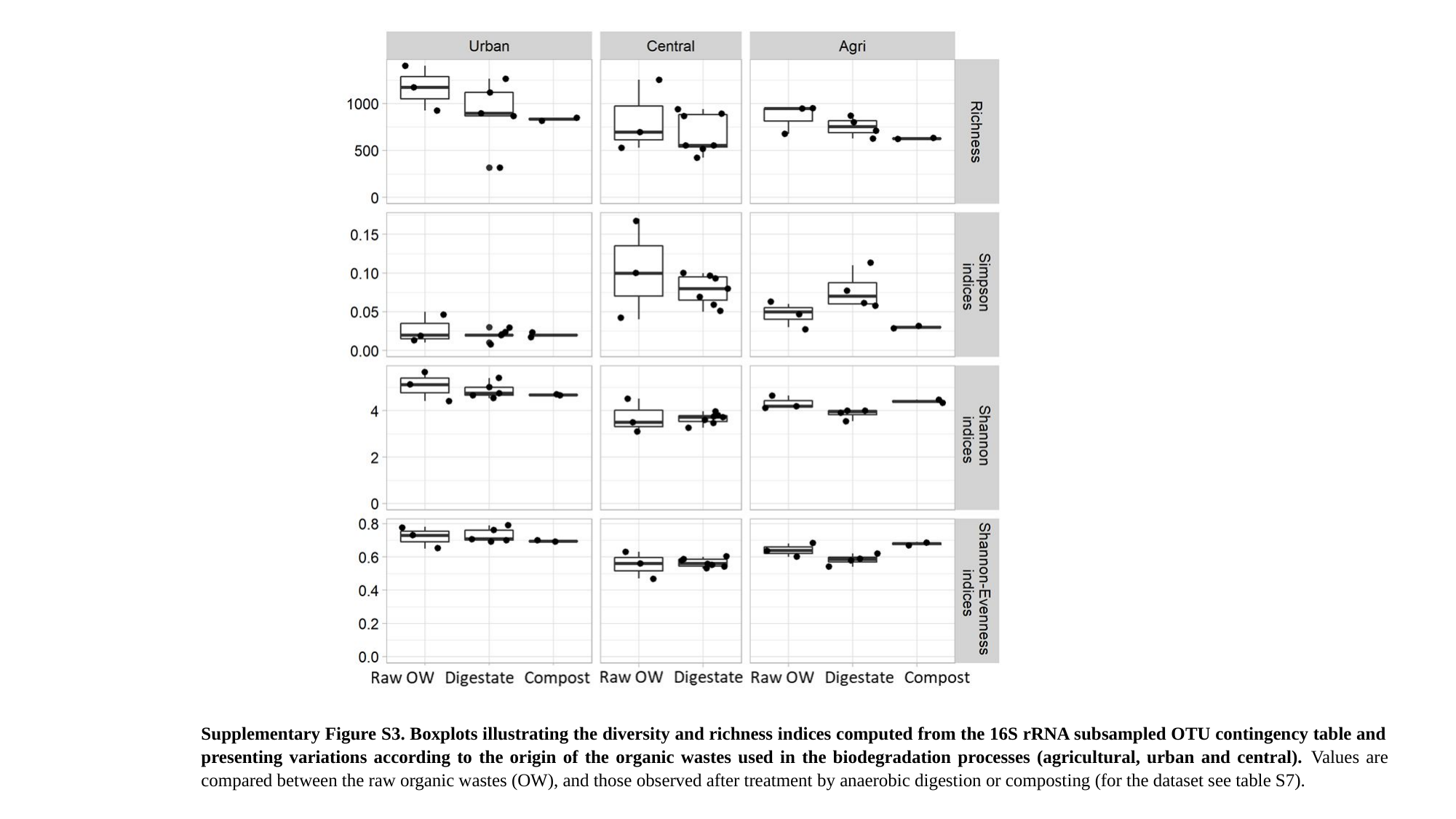

Supplementary Figure S3. Boxplots illustrating the diversity and richness indices computed from the 16S rRNA subsampled OTU contingency table and presenting variations according to the origin of the organic wastes used in the biodegradation processes (agricultural, urban and central). Values are compared between the raw organic wastes (OW), and those observed after treatment by anaerobic digestion or composting (for the dataset see table S7).

## Slide 7
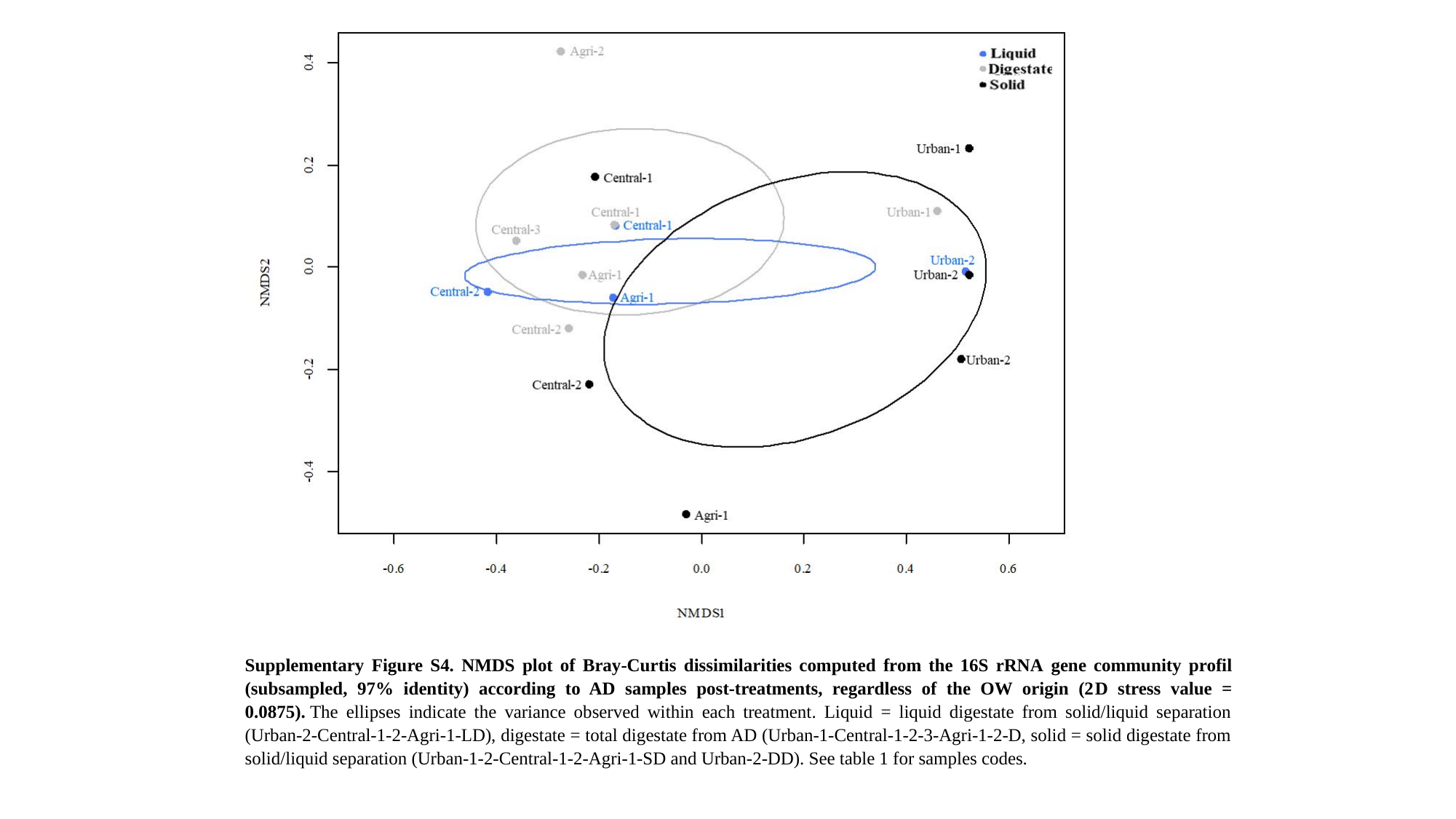

Supplementary Figure S4. NMDS plot of Bray-Curtis dissimilarities computed from the 16S rRNA gene community profil (subsampled, 97% identity) according to AD samples post-treatments, regardless of the OW origin (2D stress value = 0.0875). The ellipses indicate the variance observed within each treatment. Liquid = liquid digestate from solid/liquid separation (Urban-2-Central-1-2-Agri-1-LD), digestate = total digestate from AD (Urban-1-Central-1-2-3-Agri-1-2-D, solid = solid digestate from solid/liquid separation (Urban-1-2-Central-1-2-Agri-1-SD and Urban-2-DD). See table 1 for samples codes.
